# Measuring publication impact, and publishing and funding models

**Published:** 2016

**Authors:** Paul Brink

**Affiliations:** Department of Medicine, University of Stellenbosch, South Africa

The impact factor, or, more correctly, the journal impact factor [JIF; Thompsons Reuters (ISI)] has featured in previous reports of the Cardiovascular Journal of Africa (CVJA).^1^,^3^ As expected, it has been steadily rising and is now at 1.022 (2015). This is not to be scoffed at. Of the 14 listed medical journals in Africa, it is third to the South African Medical Journal (SAMJ; JIF = 1.5). Similarly, in another major database, Scopus, it ranks at number 184 out of 333 journals of cardiovascular medicine globally. Within Africa it is the only cardiovascular journal indexed by Thompson Reuters and also by Scopus. These statistics are based on citations to articles that appear in journals, and formulae that relate the number of citations to published articles in a journal over a given time period,^4^ and are part of the more extensive ways of evaluating scientific output under the umbrella term bibliometrics.

Historically, authors tied their status to that of the journals in which they published, and the higher the JIF, the higher the status of an author. However, similar exercises can also be performed for an article impact and individual impact (H factor). This is very nicely set out in the article by Agarwal et al., where using the first author as an example, the gamut of journal, article and individual-level citation-based statistics are calculated and discussed.^4^

However, although the journal is progressing well, there are significant challenges, and as a medico-scientific publication, the CVJA receives many more submissions than it accepts for publication. These are turbulent times in the publishing world and one is reminded of the words in Bob Dylan’s song (1964), “The times they are a-changin’, … you better start swimmin’ or you’ll sink like a stone, for the times they are a-changin’.”

Globally, the reading of printed versions of newspapers and scholarly articles has declined, particularly of the latter, in favour of the internet. However, advertisements in printed journals are a very important source of income.

Furthermore, concomitant with these changes in printdistribution has been the Open Access (OA) movement, which believes that the products of science should be freely available to all and easily attainable since publishing on the internet, superficially at least, is cheap. However, even if articles are not printed on paper, there are many processes involved in publishing. Some of these include the handling of submissions, refereeing of submitted articles (traditionally free of charge by fellow scientists), editing, proof reading, loading onto a website and management of the website. Therefore personnel and outsourcing functions become necessary.^5^

This has led to public-funded research reports, with universities and some private organisations requiring scientific content to be freely available on publication on the internet. This removes subscription incentives. In addition, being accepted into PubMed Central (PMC) also requires content to be made freely available. PMC, as pointed out in a previous article, may effectively steal traffic.^3^ Targeting articles of interest and finding them on PMC does not even bring the searcher to the website of the journal. So, this becomes a disincentive to both subscription to journals and to buying single articles.^3^

Print has rapidly been making way for a digitised form of presentation and the CVJA has kept pace with this trend.^6^ The journal has also followed suit with OA, it has been accepted by PMC, and its content is freely available in other ways.^2^,^3^ To make up for the loss of income from reducing printing, for a fee, articles are published online ahead of publication. Many authors are making use of this option, but it does not adequately cover the loss of income.

This is an appropriate time to consider the role of countrybased or territorial journals; in this case the CVJA, which is the official journal for the Pan-Africa Society of Cardiology. Important functions of a medico-scientific journal are to publish quality, peerreviewed, original scientific articles and good review articles. Credible reviews are very important as we are competing with predatory journals that solicit articles with a promise of quick (mostly superficial) reviews and acceptance of almost all submissions.^7^ Other important functions for societies are the publishing of abstracts from congresses, and providing a platform for exchange of letters, debates, dissemination of policy, guidelines, community news, and news on appliances and pharmaceutical developments.

How then should medico-scientific journals be funded in this changed environment? Most OA journals now require an articleprocessing charge (APC).^8^ This is the so-called ‘author-pay’ model. Even journals that remained propriety-based with paid-for content, such as the New England Journal of Medicine (NEJM) and Circulation, will make content available on an OA basis if a research sponsor requires it.

Consequently, we now see three types of journals in terms of availability of content, namely, pure OA, hybrid (commercial content-for-sale with some OA articles) and pure content-for-sale journals. With regard to viewing the content, we see it as pure internet, hybrid internet and paper-based content and, nowadays, the almost extinct pure paper-based distribution of content. APCs average at about US$2 000 while hybrid journals such as Circulation and NEJM average at about US$3 000 per article if it has to be OA.^8^

The CVJA currently requires a submission fee to cover at least the submission costs. This fixed fee is because every submission on the Editorial Manager system costs money, whether the article is accepted or not. Most OA journals just request an APC on acceptance of a manuscript for publishing. CVJA will have to follow this trend, in line with other journals, or the route of maintaining the submission fee, and add on an APC.
